# Smoking and alcohol drinking in relation to the risk of esophageal squamous cell carcinoma: A population-based case-control study in China

**DOI:** 10.1038/s41598-017-17617-2

**Published:** 2017-12-08

**Authors:** Xiaorong Yang, Xingdong Chen, Maoqiang Zhuang, Ziyu Yuan, Shuping Nie, Ming Lu, Li Jin, Weimin Ye

**Affiliations:** 1 Department of Epidemiology, School of Public Health, Shandong University, Jinan, China; 2 The State Key Laboratory of Genetic Engineering, Collaborative Innovation Center for Genetics and Development, School of Life Sciences, Fudan University, Shanghai, China; 3 Fudan University Taizhou Institute of Health Sciences, Taizhou, China; 4 Department of Medical Epidemiology and Biostatistics, Karolinska Institutet, Stockholm, Sweden; 5 Shandong Center for Disease Control and Prevention, Jinan, China; 6 Clinical Epidemiology Unit, Qilu Hospital of Shandong University, Jinan, China

## Abstract

Previous results regarding the associations between esophageal squamous-cell carcinoma (ESCC) risk and smoking/alcohol drinking in high-risk areas are inconsistent. We performed a large population-based case-control study from 2010 to 2013 in a high-incidence area of China, and enrolled 1353 ESCC cases and 1961 controls. Data regarding smoking and alcohol drinking were collected via face-to-face interviews using a structured questionnaire. Odd ratios (ORs) with 95% confidence intervals (CIs) were estimated using unconditional logistic regression models. After adjusting for alcohol drinking and other potential confounders, male heavy smokers (i.e., those who started smoked more than 20 cigarettes per day or 40 pack-years, or started smoking early), showed a moderately increased risk for ESCC; however, current smoking was not associated with an increased risk. Alcohol drinking among males significantly increased the risk for ESCC (OR = 2.20, 95%CI:1.79~2.70). We observed increasing excess ESCC risks with decreasing age at behavior initiation as well as with increasing duration and intensity of alcohol intake, which were particularly evident among current smokers. In contrast, neither smoking nor alcohol drinking was not associated with ESCC risk among females. In conclusion, alcohol drinking shows a monotonic dose-response relationship with ESCC risk among men, and this relationship is particularly evident among smokers.

## Introduction

According to the most recent International Agency for Research on Cancer (IARC) report, 455,800 new esophageal cancer cases (3% of all cancers) were reported and 400,200 deaths due to esophageal cancer (5% of all cancer deaths) occurred worldwide in 2012^1^. The incidence of esophageal cancer, one of the most fatal malignancies, shows a conspicuous geographical variation across the world, and approximately half of all new esophageal cancer cases occurred in China^2^. Esophageal cancer has two predominant histopathological subtypes: esophageal squamous cell carcinoma (ESCC) and esophageal adenocarcinoma (EAC). In the high-incidence area, (i.e., Central and East Asia as well as eastern Africa), 90% of esophageal cancer cases are ESCC, compared with less than 30% in North America^3^.

The leading risk factors for ESCC in Western countries are tobacco smoking and alcohol consumption which together account for almost 90% of the population attributable fraction^4,5^. However, the corresponding contribution of smoking and alcohol consumption in high-risk areas is considerably weaker and the conclusion remains controversial^1,6^. Some studies have reported that active cigarette smoking marginally affects ESCC risk, with an approximately 1.4-fold relative risk^7–11^. Other studies have shown that current smokers have a 2.5-to-4-fold relative risk of ESCC, compared with never smokers^12–15^. Few studies have explored the association between exposure to passive smoking and ESCC risk. Thus far, two available studies have indicated some adverse effects of passive smoking on the risk of ESCC^16,17^. Similarly, the results regarding the effects of alcohol intake on ESCC risk in high-risk areas are inconsistent; the reported magnitudes of these associations range from no relationship^7,8,12^ to 1.5–3.5-fold relative risk^10,13,14,18^.

Although these controversial results might partly be explained by regional particularities, certain disadvantages associated with the study designs of previous studies might be the main culprits. Usually only baseline information regarding smoking and alcohol drinking is used in cohort studies, which ignores the changes in these habits during the follow-up period^7,14^. In most case-control studies, cases are recruited weeks or months after diagnosis, which might result in recall bias because patients would ponder and overstate their exposure to potential risk factors^8,10–13^. Inadequate sample sizes also result in unstable estimates of relative risks.

Thus, we initiated a rigorously designed population-based case-control study of upper gastrointestinal cancer in Taixing, a high-incidence area in China^19^. Since more than 95% of esophageal cancer cases are ESCC in China^3^, the present analysis focused on the effects of tobacco smoking and alcohol drinking on ESCC risk in this high-risk population.

## Materials and Methods

### Study design and participants

The study design has been described in detail previously^20,21^, and the current report is based on previous materials and further extended 1.5 years. In brief, we performed a case-control study in Taixing, China, a city of approximately 1.13 million inhabitants. We limited study participants to those who were 40–85 years old and had lived in Taixing for at least 5 years. We aimed to enroll as completely as possible all newly-diagnosed esophageal cancer cases from October 2010 to September 2013. More than 90% of these patients in this area were diagnosed at the local four largest hospitals (the Taixing People’s Hospital, the Second People’s Hospital of Taixing, the Taixing Chinese Medicine Hospital and the Third People’s Hospital of Taixing). As long as a doctor suspected that a patient might have esophageal cancer during an endoscopic examination, the patient was asked to complete a questionnaire by trained interviewers. After a histopathological examination, patients who were not histopathologically confirmed were excluded from the study. To further search for esophageal cancer cases missed by endoscopy units for various reasons, we performed further searches the local Cancer Registry. At the end of each year, when the local Cancer Registry had compiled a list of incident cases, we compared their patient list with our list from the four aforementioned hospitals to identify missing cases.

**Figure 1:**
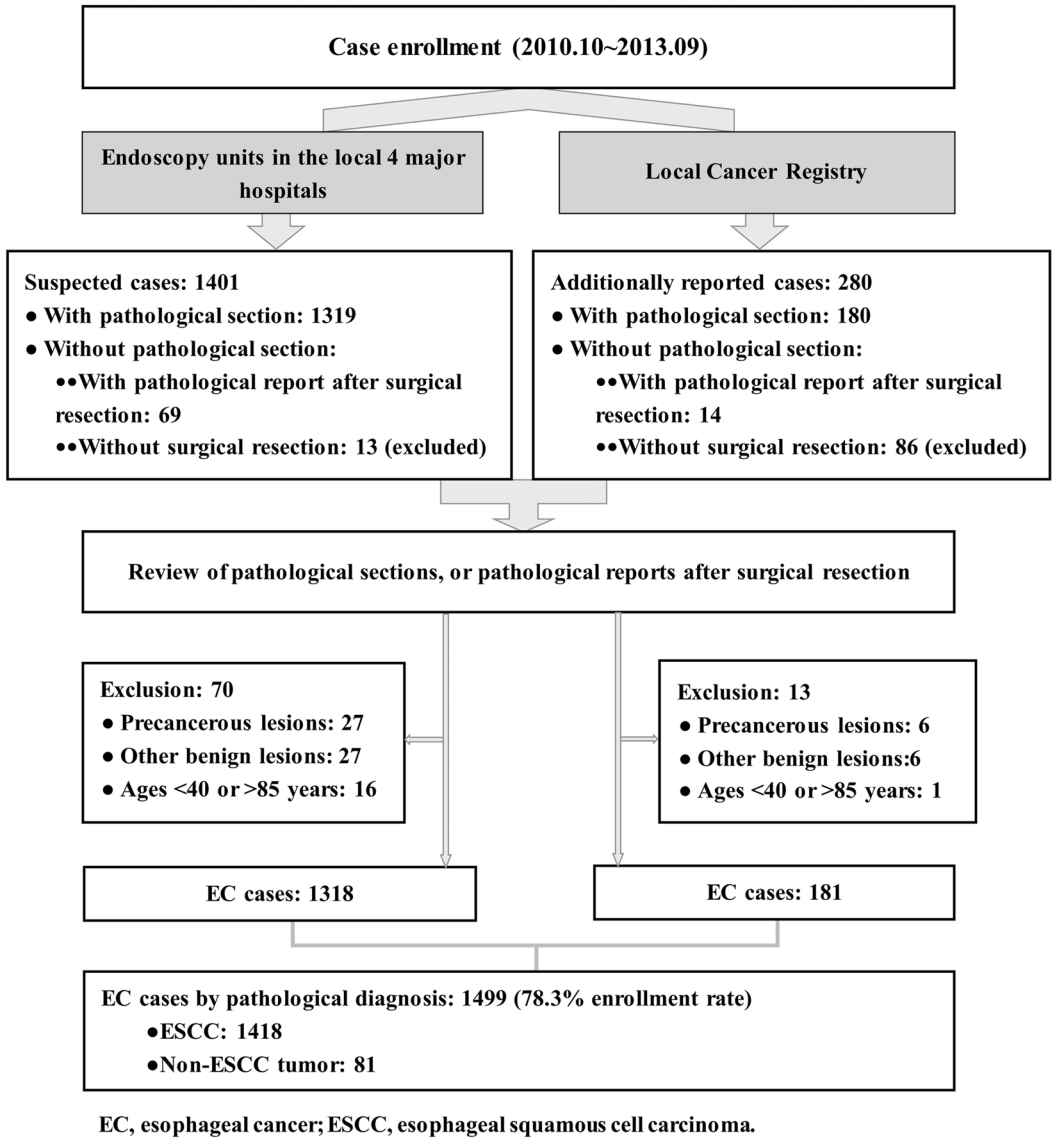
The flow chart for the recruitment of esophageal squamous cell carcinoma cases in a population-based case-control study conducted in Taixing, China.

During the three-year period, we gathered 1401 suspected cases from the hospitals’ endoscopy units. Furthermore, by linking with the local Cancer Registry, 994 potential cases were additionally identified; of these patients, 291 died before being contacted, 247 were living outside Taixing or too ill to participate and 176 refused, leaving 280 who were recruited for the study. For each case, we also attempted to collect sections from formalin-fixed and paraffin-embedded tissue blocks; for those whose tissue blocks were not available, original pathological reports after surgical resection were collected. In summary, we enrolled 1681 potential esophageal cancer cases; of these patients, 1499 suspected or reported cases provided tissue sections. Of the remaining 182 cases whose tissue blocks were not available, we gathered original pathological reports after surgical resection for 83 cases. We excluded the remaining 99 cases who were not operated upon from this study. After a review of the pathological sections by the study pathologist, and a reassessment of the surgical pathological reports for 83 cases, 1499 esophageal cancer cases (1418 cases of ESCC and 81 cases of non-ESCC) were confirmed and enrolled for this study. We estimated that approximately 78.3% of the incident cases in the study base were included based on the estimated number from the local Cancer Registry. The detailed selection flow of the esophageal cancer cases is illustrated in Fig. 1.

This upper gastrointestinal tract cancer case-control study enrolled esophageal and gastric cancer cases simultaneously. Because the age and sex distributions of these cancer cases were similar, the combined corresponding controls were randomly drawn from the general population. In brief, control participants were randomly selected every 12 months during the same period from the local Population Registry, which includes the full population of Taixing. To increase the statistical power of this study, we employed a frequency-match method for control selection, where the strata were defined by sex and 5-year age groups. For each stratum of the cases, we selected corresponding controls with a 1.3:1 ratio, considering an approximately 75% response rate among controls based on the results of a pilot study. During the 3-year period, we enrolled 2699 potential esophageal and gastric cases. Furthermore, 3501 corresponding population controls were randomly selected; of these participants, 643 were excluded due to death prior to contact, outmigration or an inability to be reached, leaving 2858 eligible participants. Finally, 2011 controls participated in this study (participation rate: 70.4%); of these controls, 19 were not in the eligible age range (40–85 years), thereby leaving 1992 controls who were recruited for the current study. Because the age and gender distributions between esophageal cases and gastric cases were consistent, the data of all enrolled controls were used in this ESCC study.

The current analysis was based on 1418 ESCC cases, which were independently reviewed and confirmed, and 1992 controls. After further excluding 96 participants with incomplete questionnaire information concerning the main exposure variables, we included 1353 cases and 1961 controls in the final analysis.

### Exposure assessment

Trained staff interviewed all participants in person using an electronic questionnaire. This structured questionnaire covers information on demographics, family wealth, family history of cancer, oral hygiene, personal medical history, smoking, alcohol and tea drinking, and dietary history.

### Smoking and drinking history

This section addresses each participant’s lifelong history of tobacco smoking and alcohol consumption up to interview date. Participants were asked at what age they started smoking/drinking, at what age they stopped smoking/drinking permanently (if an ex-smoker or ex-drinker), the amount that they smoked/drank in a typical day, the average number of days per week or per month they smoked/drank, and the number of years they had smoked/drank. In addition, information about individual household passive smoking exposure in childhood/adulthood and passive smoking exposure at work was collected, addressing smoking intensity and duration for each family member, and the average time per day and years of passive smoking exposure at work.

### Definition of smoking and alcohol drinking

Users of tobacco or alcohol were defined as those who used the respective product for at least 6 months and reached the floor level during this period (smoked at least 1 cigarette every three days/drank alcohol at least once per week). Ex-smokers and ex-drinkers were defined as those who had quit smoking tobacco or drinking alcohol at least 2 years before interview date because early symptoms of esophageal cancer might cause patients to quit smoking or drinking.

Smoking/drinking duration was defined as the time between age at starting and either permanent quitting (for ex-smokers/ex-drinkers) or the interview (for current smokers/drinkers) after subtracting the cumulative duration of any episodes of temporarily quitting. Smoking intensity was defined as the average number of cigarettes smoked per day. Drinking intensity was defined as the average amount of ethanol intake per week. We estimated each participant’s lifetime cumulative quantity of tobacco smoked in pack-years. Time since quitting was calculated as the difference between the age at which ex-smokers/ex-drinkers had permanently stopped and their age at interview. For data analysis, we categorized the derived continuous variables related to smoking and alcohol drinking using approximate quartile cut-offs from the distribution of controls, except for certain groups with actual meaning. The association between passive smoking and ESCC was analyzed only with regard to never smokers.

### Statistical methods

Because the prevalence and pattern of smoking and alcohol drinking in China are extremely different between men and women^22,23^, we analyzed data separately by sex. We used unconditional logistic regression models to estimate odds ratios (ORs) with 95% confidence intervals (CIs) for ESCC in association with smoking and alcohol drinking. Three major models were fitted: the age-adjusted model included only age (continuous variable) as a covariate, while the fully adjusted model (excluding smoking/alcohol-drinking status) included additional potential confounders, such as education, marital status, occupation, family wealth score, body mass index 10 years ago, sum of missing and filled teeth, times of tooth brushing per day, tea drinking temperature, dietary energy intake 10 years ago, and family history of esophageal cancer among first-degree relatives (categorized variables as shown in Table 1). To further control for the confounding effects of smoking and alcohol drinking, we also fitted fully adjusted models by adding the alcohol drinking variable into the model when analyzing smoking as exposure variable and vice versa. For exposure to passive smoking, we analyzed exposures to household passive smoking during childhood or adulthood, and passive smoking at work during adulthood separately. Family wealth score was calculated based on the ownership of valuable home items using a multiple correspondence analysis. These scores were categorized as quintiles according to the observed coordinates among control participants^20^. Except for smoking and alcohol drinking status variables, we performed trend tests using the median within each categorical variable, and never smokers/drinkers were used as the reference group. To test the modification of the alcohol-ESCC association by smoking, we used likelihood ratio tests for nested models with and without interaction terms. The non-linear relationship between alcohol drinking and ESCC risk was further examined using a restricted cubic spline regression with 5 knots for current and non-current smokers separately. All analyses were conducted using STATA version 13.1 (Stata Corporation, College Station, TX, USA). Two-tailed *p*-values less than 0.05 were considered significant.

**Table 1:** Demographic information of the study subjects enrolled in a case-control study on esophageal squamous cell carcinoma, Taixing, China (N = 3314).

Variables	Men (n = 2273)			Women (n = 1041)		
	Controls (n = 1352) N (%)	Cases (n = 921) N (%)	*p* value^a^	Controls (n = 609) N (%)	Cases (n = 432) N (%)	*p* value^a^
Age at interview (mean ± S.D., years)	65.5 ± 8.4	65.2 ± 8.4	0.199	67.6 ± 9.4	69.3 ± 7.6	0.032
Age at interview (years)						
40–49	52 (3.85)	30 (3.26)	0.216	29 (4.76)	5 (1.16)	<0.001
50–59	276 (20.41)	205 (22.26)		90 (14.78)	35 (8.10)	
60–69	580 (42.90)	406 (44.08)		206 (33.83)	177 (40.97)	
70–79	396 (29.29)	237 (25.73)		231 (37.93)	177 (40.97)	
80–85	48 (3.55)	43 (4.67)		53 (8.70)	38 (8.80)	
Education level						
Illiteracy	166 (12.28)	163 (17.70)	<0.001	361 (59.28)	313 (72.45)	<0.001
Primary school	577 (42.68)	401 (43.54)		169 (27.75)	102 (23.61)	
Junior school	459 (33.95)	274 (29.75)		67 (11.00)	15 (3.47)	
High school and above	150 (11.09)	83 (9.01)		12 (1.97)	2 (0.46)	
Marital status						
Unmarried	62 (4.59)	55 (5.97)	0.233	5 (0.82)	2 (0.46)	0.555
Married	1145 (84.69)	758 (82.30)		422 (69.29)	290 (67.13)	
Divorce/Widow	145 (10.72)	108 (11.73)		182 (29.89)	140 (32.41)	
Occupation						
Farmer	698 (51.63)	485 (52.66)	0.857	537 (88.18)	408 (94.44)	0.003
Worker	353 (26.11)	239 (25.95)		45 (7.39)	16 (3.70)	
Service/Clerk/Professional/Administrator	301 (22.26)	197 (21.39)		27 (4.43)	8 (1.85)	
Family wealth score						
Q1	283 (20.93)	277 (30.08)	<0.001	118 (19.38)	119 (27.55)	<0.001
Q2	233 (17.23)	162 (17.59)		118 (19.38)	88 (20.37)	
Q3	286 (21.15)	210 (22.80)		144 (23.65)	106 (24.54)	
Q4	297 (21.97)	176 (19.11)		132 (21.67)	83 (19.21)	
Q5	253 (18.71)	96 (10.42)		97 (15.93)	36 (8.33)	
Body mass index 10 years ago						
<18.5	60 (4.44)	70 (7.60)	<0.001	55 (9.03)	56 (12.96)	0.008
[18.5, 24)	847 (62.65)	622 (67.54)		335 (55.01)	241 (55.79)	
[24, 28)	363 (26.85)	189 (20.52)		173 (28.41)	121 (28.01)	
≥28	82 (6.07)	40 (4.34)		46 (7.55)	14 (3.24)	
Sum of missing and filled teeth						
None	370 (27.37)	241 (26.17)	0.195	130 (21.35)	57 (13.19)	0.001
<6	512 (37.87)	325 (35.29)		201 (33.00)	130 (30.09)	
≥6	465 (34.39)	350 (38.00)		277 (45.48)	237 (54.86)	
Missing	5 (0.37)	5 (0.54)		1 (0.16)	8 (1.85)	
Times of tooth brushing per day						
<2	897 (66.35)	745 (80.89)	<0.001	387 (63.55)	347 (80.32)	<0.001
≥2	450 (33.28)	170 (18.78)		222 (36.45)	85 (19.68)	
Missing	5 (0.37)	3 (0.33)				
Tea drinking temperature						
Never	857 (63.39)	493 (53.53)	<0.001	578 (94.91)	418 (96.76)	0.281
Mild	208 (15.38)	157 (17.05)		19 (3.12)	11 (2.55)	
Hot	207 (15.31)	169 (18.35)		8 (1.31)	1 (0.23)	
Very hot	80 (5.92)	102 (11.07)		4 (0.66)	2 (0.46)	
Dietary energy intake (Kcal/per day) 10 years ago						
<1250	572 (42.31)	439 (47.67)	0.010	396 (65.02)	303 (70.14)	0.121
≥1250	767 (56.73)	471 (51.14)		199 (32.68)	123 (28.47)	
Missing	13 (0.96)	11 (1.19)		14 (2.30)	6 (1.39)	
Family history of esophageal cancer among first-degree relatives						
No	1100 (81.36)	623 (67.64)	<0.001	496 (81.44)	294 (68.06)	<0.001
Yes	250 (18.49)	297 (32.25)		112 (18.39)	138 (31.94)	
Missing	2 (0.15)	1 (0.11)		1 (0.16)	0 (0.00)	
Smoking type						
Never	299 (22.12)	153 (16.61)	0.009	585 (96.06)	413 (95.60)	0.878
Cigarettes	1011 (74.78)	741 (80.46)		23 (3.78)	18 (4.17)	
Pipe	31 (2.29)	18 (1.95)		1 (0.16)	1 (0.23)	
Water pipe	11 (0.81)	8 (0.87)		0 (0.00)	0 (0.00)	
Hand-rolled cigarettes	0 (0.00)	1 (0.11)		0 (0.00)	0 (0.00)	
Alcohol drinking type						
Never	584 (43.20)	235 (25.52)	<0.001	574 (94.25)	400 (92.59)	0.071
Strong Chinese spirits (Median 55% ABV)	744 (55.03)	680 (73.83)		29 (4.76)	26 (6.02)	
Moderate Chinese spirits (Median 38% ABV)	10 (0.74)	5 (0.54)		1 (0.16)	6 (1.39)	
Beer	7 (0.52)	1 (0.11)		1 (0.16)	0 (0.00)	
Fruit wine	2 (0.15)	0 (0.00)		1 (0.16)	0 (0.00)	
Yellow rice wine	2 (0.15)	0 (0.00)		2 (0.33)	0 (0.00)	
Grape wine	0 (0.00)	0 (0.00)		1 (0.16)	0 (0.00)	
Imported high spirits	3 (0.22)	0 (0.00)		0 (0.00)	0 (0.00)	

S.D.: standard deviation; ABV: alcohol by volume.

^a^p values were derived using Kruskal–Wallis test for continuous variables, and Chi-squared test or Fisher exact test for categorical variables, after excluding the corresponding missing value.

^b^Combining ex-tea drinkers and current tea drinkers, because there were only 40 ex-tea drinkers among males.

### Ethical statement

The study protocol was approved by the institutional review boards of the School of Life Sciences, Fudan University and Qilu Hospital, Shandong University. This study was conducted in accordance with the approved protocol, and all participants provided written informed consent.

### Data availability

The datasets generated and analyzed during the current study are available from the corresponding authors on reasonable request.

## Results

### Demographics and exposure factors distribution

The characteristics of both control participants and ESCC cases stratified by sex are summarized in Table 1. Male cases tended to have less education, lower family wealth scores, lower body mass indices 10 years ago, less dietary energy intake 10 years ago and fewer times of tooth brushing per day. Conversely, male cases drank tea at hotter temperatures and were more likely to have family histories of esophageal cancer among their first-degree relatives. These differences were similar among women, except that female cases were slightly older than controls, more likely to be farmers, and had more missing and filled teeth. However, the differences observed among men regarding tea drinking temperature and dietary energy intake 10 years ago were not significant among women.

Of all recruited participants, more than 95% ever smokers and alcohol drinkers were men; more than 96% of smokers consumed cigarettes and approximately 97% of alcohol drinkers drank strong Chinese spirits.

### Smoking and ESCC risk

Table 2 presents the association between smoking and the risk of ESCC among men. After adjusting for age, current smokers had a close to 50% excess risk of ESCC risk compared with never smokers, whereas the OR for ex-smokers was close to unity. The excess ESCC risk associated with smoking was more evident among those who had a habit of deep inhalation during smoking, those who started smoking before age 20, those who reported a longer duration of smoking or consumed more cigarettes per day, and those who had a higher cumulative dose (pack-years) of smoking. The excess risks were partially attenuated after adjusting for other potential confounders except for alcohol drinking, but they remained largely statistically significant. After additionally adjusting for alcohol drinking, however, the excess risk was statistically significant only for those who had started smoking before age 20, those who consumed more than 20 cigarettes per day, and those who reported more than 40 smoking pack-years.

**Table 2:** The odds ratios (ORs) and 95% confidence intervals (CIs) for esophageal squamous cell carcinoma in association with smoking among males (N = 2273).

Variables	Controls N (%)	Cases N (%)	Age-adjusted OR (95%CIs)	Fully adjusted (except alcohol) OR (95%CIs)^b^	Fully adjusted OR (95%CIs)^c^
Smoking status					
Never smokers	299 (22.12)	153 (16.61)	1.00 (ref.)	1.00 (ref.)	1.00 (ref.)
Ex-smokers	153 (11.32)	81 (8.79)	1.04 (0.74~1.45)	0.95 (0.66~1.35)	0.83 (0.57~1.19)
Current smokers	900 (66.57)	687 (74.59)	1.49 (1.19~1.85)	1.32 (1.04~1.68)	1.12 (0.88~1.44)
P for trend			<0.001	0.009	0.191
Habit of deep inhalation during smoking					
No	374 (27.66)	209 (22.69)	1.09 (0.84~1.41)	0.99 (0.75~1.31)	0.86 (0.64~1.14)
Yes	679 (50.22)	559 (60.69)	1.60 (1.28~2.01)	1.42 (1.11~1.81)	1.21 (0.94~1.55)
P for trend			<0.001	0.001	0.027
Age at starting smoking (years)					
≥29	268 (19.82)	180 (19.54)	1.31 (1.00~1.72)	1.25 (0.93~1.67)	1.07 (0.80~1.44)
[23, 29)	289 (21.38)	201 (21.82)	1.35 (1.04~1.77)	1.24 (0.93~1.65)	1.07 (0.80~1.44)
[20, 23)	312 (23.08)	195 (21.17)	1.22 (0.93~1.59)	1.02 (0.77~1.37)	0.88 (0.65~1.18)
<20	184 (13.61)	192 (20.85)	2.03 (1.53~2.69)	1.76 (1.30~2.39)	1.47 (1.07~2.01)
P for trend			<0.001	0.037	0.445
Duration of smoking (years)					
≤30	254 (18.79)	176 (19.11)	1.31 (0.98~1.73)	1.23 (0.91~1.67)	1.08 (0.79~1.47)
(30, 39]	314 (23.22)	225 (24.43)	1.36 (1.05~1.78)	1.24 (0.93~1.65)	1.05 (0.78~1.41)
(39, 45]	225 (16.64)	182 (19.76)	1.58 (1.19~2.08)	1.44 (1.07~1.94)	1.20 (0.88~1.63)
>45	260 (19.23)	185 (20.09)	1.43 (1.08~1.90)	1.20 (0.88~1.62)	1.03 (0.75~1.40)
P for trend			0.001	0.066	0.584
Intensity of smoking (cig/day)					
$\le$ 10	330 (24.41)	178 (19.33)	1.05 (0.81~1.38)	0.99 (0.74~1.31)	0.85 (0.64~1.14)
(10, 20]	590 (43.64)	445 (48.32)	1.47 (1.17~1.85)	1.31 (1.02~1.68)	1.12 (0.87~1.45)
>20	131 (9.69)	140 (15.20)	2.08 (1.53~2.83)	1.84 (1.31~2.56)	1.50 (1.06~2.11)
Missing	2 (0.15)	5 (0.54)			
P for trend			<0.001	<0.001	0.005
Cumulative dose (pack-years)					
$\le$18	285 (21.08)	145 (15.74)	0.98 (0.74~1.30)	0.95 (0.71~1.28)	0.81 (0.60~1.10)
(18, 30]	237 (17.53)	181 (19.65)	1.47 (1.11~1.93)	1.32 (0.98~1.78)	1.15 (0.85~1.56)
(30, 40]	240 (17.75)	170 (18.46)	1.36 (1.03~1.80)	1.19 (0.88~1.61)	1.02 (0.75~1.38)
>40	289 (21.38)	267 (28.99)	1.81 (1.40~2.34)	1.59 (1.21~2.11)	1.34 (1.01~1.79)
Missing	2 (0.15)	5 (0.54)			
P for trend			<0.001	<0.001	0.006
Time since quitting smoking (years) among ex-smokers					
$\le$8	71 (5.25)	34 (3.69)	0.94 (0.60~1.48)	0.87 (0.53~1.43)	0.84 (0.50~1.40)
>8	82 (6.07)	47 (5.10)	1.09 (0.72~1.64)	0.89 (0.56~1.40)	0.82 (0.51~1.32)

^a^The reference group for all comparisons is never smokers.

^b^Adjusted for age (continuous), education, marital status, occupation, family wealth score, body mass index 10 years ago, sum of missing and filled teeth, times of tooth brushing per day, tea drinking temperature, dietary energy intake 10 years ago, and family history of esophageal cancer among first-degree relatives (except age, other variables are categorized as shown in Table 1).

^c^Additionally adjusted for alcohol drinking (never/ex/current alcohol drinkers).

The proportion of ever smoking among women was 4.13% (43/1041), and the majority of female smokers started smoking after age 29 (35/43), smoked fewer than 31 years (27/43), consumed no more than a half pack of cigarettes per day (25/43), and reported fewer than 17 pack-years of smoking (27/43). The fully adjusted OR for ESCC in relation to smoking among women was 1.02 (95%CI: 0.51~2.03).

The relationship between exposure to passive smoking and ESCC risk among male and female never smokers is displayed in Supplementary Table 1. We did not observe any significant association between either exposure to household passive smoking during childhood (≤18 years old) or adulthood or exposure to passive smoking at work and the risk of ESCC among either male or female never smokers.

### Alcohol drinking and ESCC risk

Table 3 shows the relative risks of ESCC in relation to alcohol drinking among men. After adjusting for age, no significant association was found among alcohol ex-drinkers compared with never drinkers, whereas current alcohol drinkers had a more than two-fold increased risk of ESCC (OR = 2.32, 95%CI: 1.92~2.79). The excess risk of ESCC increased monotonically with decreasing age at starting alcohol drinking as well as with increased duration and intensity of alcohol drinking (all *p-*values < 0.001). The results remained almost unchanged after further adjusting for potential confounders, regardless of smoking status.

**Table 3:** The odds ratios (ORs) and 95% confidence intervals (CIs) for esophageal squamous cell carcinoma in association with alcohol drinking among males (N = 2273).

Variables	Controls N (%)	Cases N (%)	Age-adjusted OR (95%CIs)	Fully adjusted (except smoking) OR (95%CIs)^b^	Fully adjusted OR (95%CIs)^c^
Alcohol drinking					
Never drinkers	584 (43.01)	235 (25.52)	1.00 (ref.)	1.00 (ref.)	1.00 (ref.)
Ex-drinkers	73 (5.23)	40 (4.34)	1.36 (0.90~2.06)	1.44 (0.92~2.25)	1.51 (0.96~2.38)
Current drinkers	695 (51.79)	646 (70.14)	2.32 (1.92~2.79)	2.27 (1.85~2.79)	2.24 (1.82~2.76)
*P* for trend			<0.001	<0.001	<0.001
Age at starting drinking (years)					
≥38	201 (14.87)	113 (12.27)	1.39 (1.05~1.83)	1.39 (1.04~1.87)	1.39 (1.04~1.87)
[28, 38)	195 (14.42)	174 (18.89)	2.23 (1.73~2.88)	2.24 (1.70~2.96)	2.23 (1.69~2.95)
[21, 28)	194 (14.35)	210 (22.80)	2.74 (2.13~3.52)	2.74 (2.09~3.60)	2.72 (2.07~3.59)
<21	178 (13.17)	189 (20.52)	2.68 (2.07~3.47)	2.64 (1.99~3.49)	2.63 (1.98~3.50)
P for trend			<0.001	<0.001	<0.001
Duration of drinking (years)					
≤25	200 (14.79)	126 (13.68)	1.55 (1.18~2.03)	1.55 (1.16~2.07)	1.56 (1.17~2.09)
(25, 34]	207 (15.31)	199 (21.61)	2.35 (1.83~3.03)	2.36 (1.80~3.09)	2.34 (1.78~3.08)
(34, 41]	172 (12.72)	165 (17.92)	2.36 (1.81~3.07)	2.30 (1.73~3.06)	2.29 (1.72~3.06)
>41	189 (13.98)	196 (21.28)	2.61 (2.02~3.37)	2.62 (1.98~3.47)	2.60 (1.96~3.46)
*P* for trend			<0.001	<0.001	<0.001
Intensity of drinking (g/day)					
≤40	180 (13.31)	133 (14.44)	1.84 (1.40~2.41)	1.93 (1.44~2.57)	1.93 (1.44~2.58)
(40, 80]	193 (14.28)	156 (16.94)	2.01 (1.55~2.61)	2.02 (1.53~2.68)	2.01 (1.52~2.68)
(80, 135]	203 (15.01)	200 (21.72)	2.46 (1.92~3.15)	2.28 (1.74~2.98)	2.27 (1.73~2.98)
>135	192 (14.20)	197 (21.39)	2.56 (1.99~3.30)	2.57 (1.95~3.39)	2.55 (1.92~3.37)
P for trend			<0.001	<0.001	<0.001
Time since quitting drinking (years) among alcohol ex-drinkers					
≤7	36 (2.66)	20 (2.17)	1.38 (0.78~2.44)	1.44 (0.79~2.66)	1.55 (0.83~2.91)
>7	37 (2.74)	20 (2.17)	1.25 (0.71~2.21)	1.55 (0.82~2.93)	1.63 (0.86~3.12)

^a^The reference group for all comparisons is never alcohol drinkers.

^b^Adjusted for age (continuous), education, marital status, occupation, family wealth score, body mass index 10 years ago, sum of missing and filled teeth, times of tooth brushing per day, tea drinking temperature, dietary energy intake 10 years ago, and family history of esophageal cancer among first-degree relatives (except age, other variables are categorized as shown in Table 1).

^c^Additionally adjusted for smoking status (never/ex/current smokers).

The proportion of alcohol drinkers among women was 6.44% (67/1041), and the majority of female alcohol drinkers started drinking after age 38 (50/67), drank alcohol fewer than 25 years (46/67), and consumed fewer than 50 g of ethanol per day (44/67). The fully adjusted OR for ESCC associated with alcohol drinking among women was 1.26 (95%CI: 0.72~2.18).

### Interaction between smoking and alcohol drinking

We performed an analysis of the joint effects of smoking and drinking on ESCC risk. Compared with non-current smokers and never alcohol drinkers, the fully adjusted ORs for current smokers but never alcohol drinkers, never smokers but ever alcohol drinkers, and both current smokers and ever drinkers were 0.82 (95%CI: 0.59~1.15), 1.41 (95%CI: 1.00~1.98) and 2.18 (95%CI: 1.64~2.92), respectively (*p-*value of interaction = 0.003).

**Figure 2:**
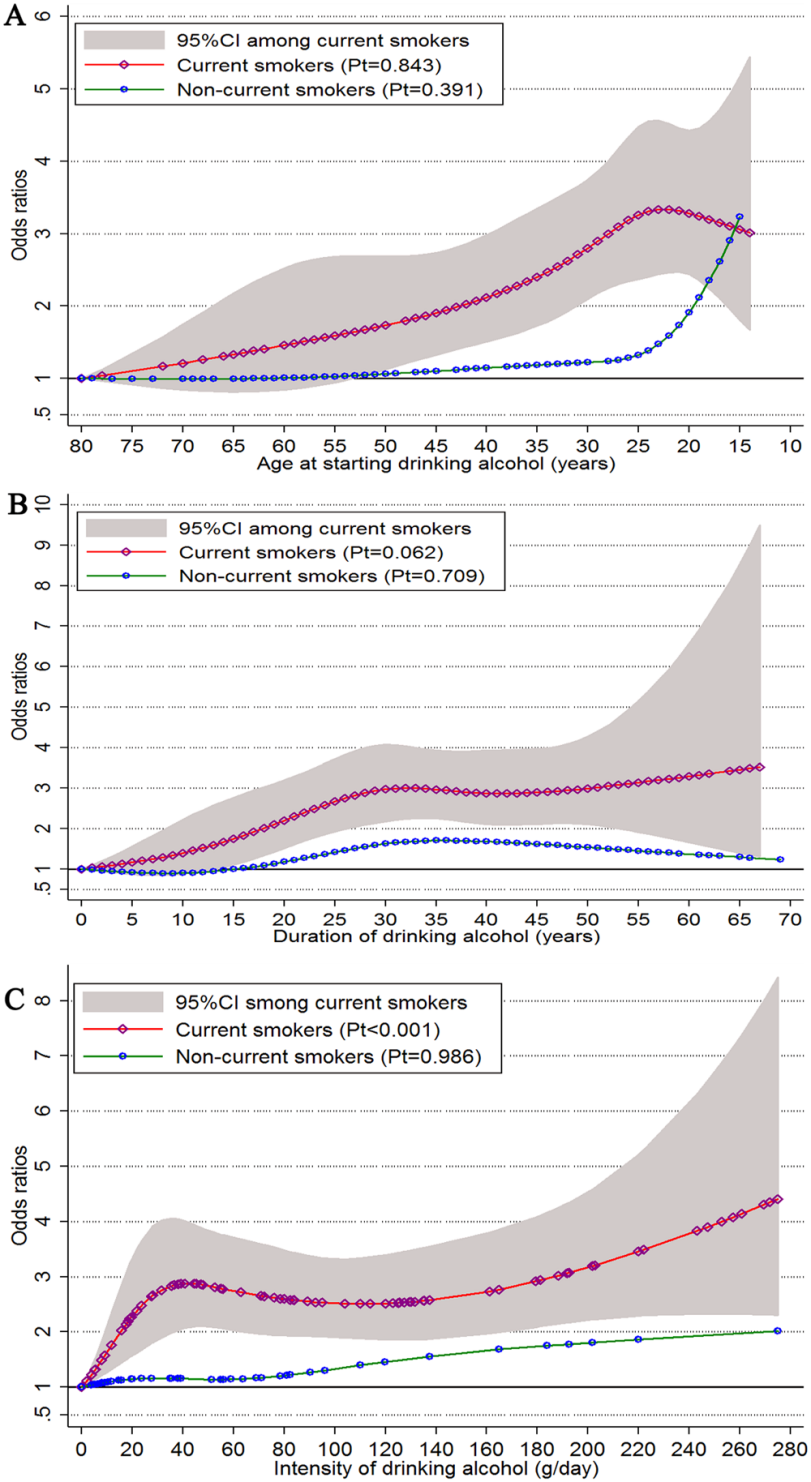
The associations between different alcohol drinking dimensions and esophageal squamous cell carcinoma with regarding to current smokers and non-current smokers separately. (**A**) age at starting drinking alcohol (years); (**B**) duration of drinking alcohol (years); (**C**) average alcohol intake (g/day). Pt, *p*-value of linear test.

Figure 2 presents the results of the restricted cubic regression analysis for alcohol intake in association with ESCC risk among current and non-current smokers, separately. The excess risks for ESCC monotonically increased among current smokers with decreasing age at starting alcohol drinking, and plateaued at around 25 years or younger. Among non-current smokers, however, almost no excess risk was observed until around age 25, and then excess risk increased steeply and reached the level of current smokers at the youngest age of starting alcohol drinking (Fig. 2A). Regarding the duration of alcohol drinking, the ORs of current smokers increased monotonically until around 3 at a duration of around 30 years. After that, excess risk increased slowly. Conversely, only modestly increased risks were noted among non-current smokers (Fig. 2B). In addition, the adjusted ORs increased sharply to around 3.0 among current smokers with light-to-moderate drinking intensity (1–40 g/day). Then the excess risk increased slowly and finally reached 4.2 at the highest level of alcohol intake (275 g/day). Among non-current smokers, however, the excess risk associated with drinking alcohol intensity increased slowly, showing a close to linear relationship, and the OR reached 2 at the highest intake level (275 g/day; Fig. 2C).

### Sensitivity analysis

We conducted a sensitivity analysis by excluding 126 male cases and 33 female cases identified in only the local Cancer Registry. The results did not change substantially. For example, compared with never smokers, the fully-adjusted OR for ESCC in relation to consuming more than one pack of cigarettes per day was 1.48 (95%CI: 1.04~2.12) in men. Compared with never alcohol drinkers, current alcohol drinkers had a more than two-fold increased risk of ESCC (fully adjusted OR = 2.40, 95%CI: 1.92–2.99).

## Discussion

Using this large population-based case-control study, we explored the associations between the risk of ESCC and various dimensions of active smoking, passive smoking, and alcohol drinking among a high-risk Chinese population. Our results indicated that smoking, especially tobacco exposure at a young age, or with a high intensity or accumulative consumption, had a marginally adverse effect on ESCC risk. However, the smoking-ESCC association was not found among women. Similarly, exposure to passive smoking was not associated with ESCC risk among either male or female never smokers. Alternatively, alcohol drinking showed a monotonic dose-response relationship with the increased risk of ESCC in men, which was pronounced among current-smokers. Alcohol drinking is not common among Chinese women, and we did not observe any association between ESCC risk and alcohol drinking among the female study population.

Our study had several strengths. To decrease the potential influence of recall bias, we interviewed the majority of cases before they were aware of their diagnosis. Other strengths of our study include the relatively large sample size, the independent verification of a case diagnosis by a study pathologist, the relatively high response rates for both cases and controls, and the systematic collection of lifelong and detailed smoking and alcohol drinking data. Importantly, we randomly selected control participants from the local Population Registry. Thus, the prevalence and pattern of smoking and alcohol drinking data of our control participants are consistent with the values reported by several large cross-sectional surveys of China^22–24^.

Despite these advantages, several study limitations should also be mentioned. First, despite our efforts to recruit study participants, non-respondents comprised 20–30% of those contacted. Thus selection bias might be a concern. However, no significant differences were found between respondents and non-respondents with regard to age and sex. Second, related information, such as flush response to alcohol drinking, which might modify alcohol’s effect on ESCC risk, was not collected. Moreover, similar to other case-control studies, recall bias might still exist despite the previously mentioned efforts to interview cases as early as possible. Therefore, we performed a sensitivity analysis by excluding those cases identified only by the local Cancer Registry, who were then interviewed after diagnosis or even treatment. The similar results partially allayed our concerns of recall bias. Finally, because of the low rates of smoking and alcohol drinking among Chinese women, our sample size was not large enough to explore the tobacco/alcohol-ESCC association in female population.

The relation between tobacco smoking and ESCC risk in China has been explored in many previous studies. Two population-based case-control studies did not find any significant association between smoking and esophageal cancer risk^16,17^. However, two cohort studies and one population-based case-control study showed that current smokers have a less than 50% excess risk of esophageal cancer^7,11,25^. Most population-based case-control studies have reported that current smokers are at a 1.7-to-2.4-fold increased risk of esophageal cancer^10,12,26,27^. Our study results are consistent with the previous studies showing that current smoking has only a marginally adverse effect on ESCC occurrence, and this effect, if one exits, might only concern heavy smokers or those who started smoking at a young age.

To the best of our knowledge, only two case-control studies have explored exposure to passive smoking and ESCC risk, and the results showed that passive smoking (OR ≈ 2.0), not active smoking, is a risk factor for ESCC in China^16,17^. A case-control study conducted in India also indicated that passive smoking exposure might increase the risk of ESCC^28^. Our study did not observe any significant association between the risk of ESCC and exposure to household passive smoking during childhood or adulthood or exposure to passive smoking at work among either male or female never smokers. Given the weak effect of active smoking observed in our study, it is reasonable to assume that passive smoking had a weak or negligible effect on ESCC in our sample. On the other hand, because of the difficulty associated with reporting exact information regarding passive smoking in the household or at work, exposure misclassification might bias any true effect towards null.

Regarding the association between alcohol drinking and esophageal risk, neither three population-based case-control studies nor one cohort study conducted in high-incidence areas of China observed any significant association^7,11,12,16^. Four other population-based case-control studies and one cohort study, however, reported that current alcohol drinkers were at an approximate 1.5-to-2.5-fold relative risk for esophageal cancer^10,17,25–27^. Although some studies have reported that modest alcohol intake (<10 g/day) is associated with a reduced ESCC risk^5,29^, the alcohol drinkers in our study usually consumed at least 50 g of alcohol, and the most common alcoholic beverage was strong Chinese liquor. Thus, we were unable to examine the effect of modest alcohol intake on ESCC risk in our study. Consistent with the results of several previous studies, our study revealed that among men, current alcohol drinkers were more than twice as likely as alcohol abstainers to develop ESCC. The excess risks of ESCC increased monotonically with decreasing age at starting alcohol consumption as well as increased duration and intensity of alcohol drinking. Furthermore, we observed that smoking strongly modified the alcohol-ESCC association, which is consistent with the reports of several previous studies^10,25,26,29^.

Our study indicates that alcohol intake, especially when combined with tobacco smoking, significantly increases the risk of ESCC. The general molecular mechanism of the carcinogenesis effects of ethanol and cigarette smoke has been elaborated upon in a previous review^30^. Acetaldehyde, an ethanol metabolite, plays the most important role in promoting carcinogenesis, while ethanol itself inhibits DNA methylation and interacts with retinoid metabolism. More than 60 carcinogens in cigarette smoke are strongly linked to smoking-induced carcinogenesis^30^. However, the differential tissue distribution of adverse substances and their metabolites might determine the observed difference between alcohol-ESCC and smoking-ESCC associations. Because the recent molecular classification of ESCC indicates the subtypes of ESCC show different geographic distributions^31^, the different effects of smoking and alcohol drinking for ESCC incidence between high-risk areas and low-risk areas might result from specific molecular pathways.

The incidence rate of ESCC among men is 2–4 times higher than that in women in China^2^, and the male-to-female ratio among our recruited ESCC cases was 2.13. In our study, alcohol drinking and smoking were much more common among men, which might explain the major sex difference in the ESCC incidence in China to a large extent.

In summary, alcohol drinking, especially in combination with tobacco smoking, significantly increases the risk of ESCC among Chinese men. This evidence highlights the importance of integrating the elimination of these modifiable lifestyle risk factors into primary prevention strategies for reducing the ESCC incidence among Chinese men. Studies with larger sample sizes or meta-analysis analysis of existing studies are needed to further explore the roles of alcohol drinking, tobacco smoking, or both in the ESCC carcinogenesis of Chinese women.

## Electronic supplementary material

Supplementary Table 1
