# Supplementary material for: Smoking and alcohol drinking in relation to the risk of esophageal squamous cell carcinoma: A population-based case-control study in China
Source: Sci Rep. 2017 Dec 8;7:17249. doi: 10.1038/s41598-017-17617-2 (PMC5722909; doi:10.1038/s41598-017-17617-2)
Supplement: Supplementary file 1 — Supplementary Table 1 [file 41598_2017_17617_MOESM1_ESM.doc]

**Smoking and alcohol drinking in relation to the risk of esophageal squamous cell carcinoma: a population-based case-control study in China**

**Xiaorong Yang^1,4^, Xingdong Chen^2,3,4^, Maoqiang Zhuang^5^, Ziyu Yuan^2,3^, Shuping Nie^6^, Ming Lu^1,3,6,*^, Li Jin^2,3^, Weimin Ye^3,4,*^**

| **Supplementary Table 1** The odds ratios (ORs) and 95% confidence intervals (CIs) for esophageal squamous cell carcinoma in association with passive smoking, among male and female never smokers (N=1450). | | | | | | |
| --- | --- | --- | --- | --- | --- | --- |
|  | Men (n=452) | |  | | Women (n=998) | |
|  | Controls/Cases | OR (95%CIs)^a^ | | Controls/Cases | | OR (95%CIs)^a^ |
| Exposure to household passive smoking during childhood | | | |  | |  |
| No | 198/100 | 1.00 (ref.) | | 366/283 | | 1.00 (ref.) |
| Yes | 101/53 | 1.31 (0.82~2.09) | | 219/130 | | 0.84 (0.63~1.12) |
| Exposure to household passive smoking during adulthood | | | |  | |  |
| No | 265/135 | 1.00 (ref.) | | 253/216 | | 1.00 (ref.) |
| Yes | 34/18 | 1.38 (0.69~2.76) | | 332/197 | | 0.76 (0.57~1.02)^b^ |
| Exposure to passive smoking at work during adulthood | | | |  | |  |
| No | 224/125 | 1.00 (ref.) | | 526/386 | | 1.00 (ref.) |
| Yes | 75/28 | 0.79 (0.45~1.41) | | 59/27 | | 0.83 (0.50~1.39) |
| ^a^ Adjusted for age (continuous), education, marital status, occupation, family wealth score, body mass index 10 years ago, sum of missing and filled teeth, times of tooth brushing per day, tea drinking temperature, dietary energy intake 10 years ago, and family history of esophageal cancer among first-degree relatives, alcohol drinking (except age, other variables are categorized as shown in Table 1).  ^b^ Additionally adjusted for husband’s occupation and education. | | | | | | |
